# Comparison of the Level and Mechanisms of Toxicity of Nanoparticles of Underwater Welding in Bioassay with Three Marine Microalgae

**DOI:** 10.3390/nano15070518

**Published:** 2025-03-29

**Authors:** Konstantin Yu. Kirichenko, Konstantin S. Pikula, Vladimir V. Chayka, Alexander V. Gridasov, Igor A. Vakhniuk, Vladislava N. Volkova, Anton V. Pogodaev, Sergei G. Parshin, Yulia S. Parshina, Yuri E. Kalinin, Aleksei S. Kholodov, Sergey M. Ugay, Tatyana Yu. Orlova, Kirill S. Golokhvast

**Affiliations:** 1 Siberian Federal Scientific Centre of Agrobiotechnologies of the Russian Academy of Science, 630501 Krasnoobsk, Russia; kirichenko@sfsca.ru (K.Y.K.); chayka@sfsca.ru (V.V.C.); vakhniuk@sfsca.ru (I.A.V.); kalininye@sfsca.ru (Y.E.K.); ugaism@sfsca.ru (S.M.U.); golokhvast@sfsca.ru (K.S.G.); 2 Polytechnic Institute (School), Far Eastern Federal University, Sukhanova 8, 690950 Vladivostok, Russia; pikula_ks@dvfu.ru (K.S.P.); gridasov.av@dvfu.ru (A.V.G.); volkova.vn@dvfu.ru (V.N.V.); pogodaev.av@dvfu.ru (A.V.P.); 3 Faculty of Biology, Peter the Great St. Petersburg Polytechnic University, 195251 St. Petersburg, Russia; parshin@spbstu.ru (S.G.P.); st068842@student.spbu.ru (Y.S.P.); 4 Far East Geological Institute, Far Eastern Branch, Russian Academy of Sciences, 159, Prospekt 100-letiya, 690022 Vladivostok, Russia; 5 National Scientific Center of Marine Biology, Far Eastern Branch, Russian Academy of Sciences, Palchevsky 17, 690041 Vladivostok, Russia; torlova06@mail.ru; 6 Advanced Engineering School “Agrobiotek”, Tomsk State University, Lenina Av., 36, 634050 Tomsk, Russia

**Keywords:** nanomaterial cytotoxicity testing, nanosafety, underwater welding, nanoparticles, oxides of metals, microalgae, underwater cutting, phytoplankton

## Abstract

In this work, the toxicity level of nano- and microparticles obtained by underwater welding was assessed. The toxicity of nano- and microparticles obtained by underwater welding was evaluated on three types of marine microalgae: *Heterosigma akashiwo* (Ochrophyta), *Porphyridium purpureum* (Rhodophyta), and *Attheya ussuriensis* (Bacillariophyta). The aim was to study the environmental risks associated with the ingress of micro- and nanoparticles of metal oxides into the marine environment. Water samples containing suspensions from wet welding and cutting processes were analyzed by inductively coupled plasma mass spectrometry (ICP-MS) to determine heavy metal concentrations. Biotesting included evaluation of growth inhibition, cell size change, and membrane potential of microalgae using flow cytometry. The results showed that samples APL-1 and APL-2 (flux-cored wire) were the most toxic, causing concentration-dependent growth inhibition of *H. akashiwo* and *A. ussuriensis* (*p* < 0.0001) as well as membrane depolarization. For *P. purpureum*, ELc and ELw (coated electrodes) samples stimulated growth, indicating species-specific responses. The stability of the nanoparticles and their bioavailability were found to play a key role in the mechanisms of toxicity. The study highlights the need to control the composition of materials for underwater welding and to develop environmentally friendly technologies. The data obtained are important for predicting the long-term effects of pollution of marine ecosystems by substances formed during underwater welding.

## 1. Introduction

Oil pipelines, both onshore and offshore, transport more than 90% of the oil produced in Russia and more than 70% worldwide [1]. In order to reduce the impact on the aquatic ecosystem, new methods of constructing underwater crossings have been widely introduced, as the trench method has a negative impact on the aquatic environment during all production processes.

One of the most important technological processes is underwater welding due to its economic efficiency and cost reduction for dry docks, etc. An integral characteristic of this process is the generation of nanoparticles [2,3,4] and their discharge into the world’s oceans. The chemical composition of these particles depends on the elements to be welded, but they are mostly metal oxides.

The properties of nanoparticles in water, such as dissolution, aggregation, and ion release, certainly influence their environmental risks [5]. It is known that particle size reduction leads to significant changes in the physicochemical and structural properties of particles, such as their surface/volume ratio, solubility, and charge distribution. These properties of nanoparticles influence their behavior in the environment and increase bioavailability and toxic effects. In particular, the stability of metal oxide nanoparticles in the biological environment, such as their ability to oxidize, reduce, and dissolve, will strongly influence their toxicity [6,7]. The relationship between the stability of nanoparticles and toxicological hazard is a matter of scientific interest [8,9] but needs additional research.

Modern researchers use various live test objects to assess the negative toxicological effects of metal oxides, including their nanoparticles. Each of these in vivo models has its own advantages and limitations. In parallel, the subsequent prognostic and translational value for humans increases as the selected organism becomes more complex. The main limitations are the ever-increasing cost and time of testing, as well as increasingly stringent regulatory and ethical restrictions [10].

We have chosen microalgae as the object of study. The reason for this choice is that changes in primary producers in the marine ecosystem, including phytoplankton and algae, lead to imbalances in the entire food chain and ecosystem, ultimately affecting nutrient recycling and waste decomposition [11]. Microalgae occupy a key trophic level as they are primary producers at the base of the aquatic food chain. They are also well suited for ecotoxicological testing because they are easy to cultivate and sensitive to pollutants. Microalgae bioassays are widely used to assess the potential toxicity of various persistent toxic substances in environmental samples due to many advantages including high sensitivity, short test duration and cost effectiveness. Microalgal bioanalysis is gradually developing, and its application to environmental samples is also expanding [12]. However, the sensitivity of different species to the same chemicals can vary widely [13].

Therefore, research on the safety and (eco)toxicity of nanoparticles is extremely important to minimize potential impacts and to support sustainable development. This work focuses on assessing the toxicological effects of underwater welding particles on living test objects.

## 2. Materials and Methods

### 2.1. Seawater Samples for Underwater Welding

In order to assess the toxicological effects on the test objects, samples were taken during underwater works with technological processes: underwater wet welding and underwater wet cutting (Table 1). The materials used for welding were 2 mm diameter flux-cored wire for cutting, 1.6 mm diameter flux-cored wire for welding, 8 mm diameter tubular exothermic electrodes for cutting, and 4 mm diameter coated electrodes for welding. Welding and cutting were performed on steel plates St3sp 300 mm long, 250 mm wide, and 10 mm thick according to GOST 16523-97. For underwater welding and cutting, we used an organic glass tank measuring 0.82 × 0.46 × 0.38 m, with a wall thickness of 1 cm. The volume of filling with sea water was 100 L.

A VD-309 P welding rectifier was used for working with electrodes.

For work with flux-cored wires, we used the complex for underwater wet welding KOPS-M (technical specifications 3441-002-83763787-2016) and welding source Svarog-500. When welding with flux-cored wire of 1.6 mm diameter, reverse current polarity was used (positive pole on the wire) and when cutting metal, direct current polarity was used (negative pole on the wire). The wire feed speed on the complex was 10 m/min. The amperage of the inverter was 400–450 A. The voltage was 46 V.

Dissolved oxygen plays a critical role in marine ecosystems. It is important to note that the concentration of dissolved oxygen in the sea constantly fluctuates throughout the year, month, and even day. This is a natural process, but the main point is that the level does not fall below the critical level of 4 mg/l, necessary to maintain the life of marine organisms. In order to study the effect of welding processes on marine biota, the results of dissolved oxygen in this process were obtained.

For reference, Table 2 shows the concentrations of individual chemical elements that we have determined in previous studies [14]. These elements were selected because they pose the greatest potential threat to living organisms. Heavy metal compounds (e.g., Cr, Zn, Ti) are highly toxic to living organisms, and those considered essential may be toxic when present in high concentrations [15]. The susceptibility of microalgae to contaminants depends on several factors, including cell size, cell wall type and thickness, and taxonomic group characteristics [16].

### 2.2. Microalgae Culture

Three types of marine microalgae—specifically, the golden-brown algae *Heterosigma akashiwo* (Hada) Hada ex Y.Hara & M.Chihara, 1987 (Ochrophyta), the red algae *Porphyridium purpureum* (Bory de Saint-Vincent) Drew et Ross, 1965 (Rhodophyta), and the diatom *Attheya ussuriensis* Stonik, Orlova et Crawford, 2006 (Bacillariophyta)—were initially isolated from Peter the Great Bay in the Sea of Japan, located in Far Eastern Russia. These microalgae were supplied by the Marine Biobank Resource Collection of the National Scientific Center of Marine Biology, part of the Far Eastern Branch of the Russian Academy of Sciences (NSCMB FEB RAS). Microalgae were selected as a model due to their role as sensitive bioindicators [17] and their importance as primary producers of organic matter in aquatic ecosystems, forming the foundation of aquatic food chains [18]. The species were chosen for their prevalence in the Sea of Japan [19] and their established use as test organisms in ecotoxicological studies [20,21].

The cultivation of microalgae and the conditions for toxicity testing were conducted in accordance with the guidelines outlined in OECD No. 201 [22], with slight modifications as described below. The microalgae were grown using Guillard’s f/2 medium [23]. For the experiments, filtered (using a filter with a pore diameter of 0.22 µm) and sterilized seawater with a salinity of 33 ± 1‰ and a pH of 8.0 ± 0.2 was utilized. The cultivation process was maintained at a temperature of 20 ± 2 °C, with a light intensity of 300 µmol photons/m^2^/s and a light-to-dark cycle of 12:12 h. All bioassays were conducted under these identical conditions.

Prior to the experiment, microalgae cells were grown in 250 mL Erlenmeyer flasks. For the bioassays, algal cultures in the exponential growth phase were selected. The starting cell density for the bioassays was set at 2 × 10^4^ cells per mL for *H. akashiwo* and *A. ussuriensis*, while for *P. purpureum*, the initial cell density was 6 × 10^4^ cells per mL.

### 2.3. Experimental Design and Sample Preparation

The welding samples were evaluated using a microalgae growth rate inhibition test conducted in 24-well plates. Each well contained 500 µL of stock microalgae aliquots and 1500 µL of the test sample. In the control group wells, 500 µL of microalgae aliquots were mixed with 1500 µL of filtered seawater. For the treated wells, 500 µL of stock microalgae aliquots were combined with 1500 µL of a mixture containing the welding suspension and seawater, resulting in final exposure concentrations of 10%, 25%, 50%, and 75% of the welding suspension. Each concentration, along with the control group, was tested in quadruplicate. After 72 h of exposure, cell counts were performed using a CytoFLEX flow cytometer (Beckman Coulter, Indianapolis, IN, USA). The protocol for cell counting and the subsequent processing of the results is detailed in the following section.

### 2.4. Flow Cytometry: Cell Count, Staining Protocols, and Post Processing

The CytoFLEX flow cytometer with the CytExpert v.2.5 software package was utilized to assess growth rate inhibition, cell size, and changes in membrane potential of microalgae cells following exposure. Fluorescent dyes were employed to differentiate between live and dead microalgae cells and to analyze biochemical alterations in the exposed cells. All measured endpoints and the parameters for their detection are outlined in Table 2. In all instances, a blue laser (488 nm) from the CytoFLEX flow cytometer served as the excitation source. The excitation source and emission channels were chosen based on the maximum emission wavelengths of the fluorescent dyes, as specified by the manufacturer (Molecular Probes, Eugene, OR, USA). The optimization of dye concentrations and staining duration, as described in our previous study [4], was re-evaluated prior to each measurement series (Table 3). The staining duration for all dyes and microalgae species was set at 20 min.

The assessment of microalgae growth rate inhibition was conducted through direct cell counting. Microalgae cells were identified based on their size and granularity, as visualized in dot cytograms generated from forward and side scattering of the blue laser, as well as by the fluorescence of chlorophyll *a* (detected at an emission channel of 690 nm). To ensure accurate counts, dead cells were excluded by staining the samples with propidium iodide (PI) following a standard bioassay protocol [24]. PI functions by intercalating between DNA or RNA base pairs, which results in a 20–30-fold increase in its fluorescence intensity [25]. Since PI cannot penetrate the intact membranes of living cells, only cells exhibiting significantly elevated fluorescence intensity at the 610 nm emission filter were considered dead and excluded from the cell count.

In all assays, each sample was analyzed at a flow rate of 50 µL/min for a duration of 30 s. During cell counting, the number of cells in the control group was normalized to 100% as the baseline reference.

To measure the size of microalgae cells, a size calibration kit (batch F13838, Molecular Probes, Eugene, OR, USA) with certified size distributions of 1, 2, 4, 6, 10, and 15 μm was utilized for the forward scatter emission channel. The distribution of cells in the control group across these size ranges was normalized to 100% as the reference baseline.

The membrane potential of microalgae cells was evaluated using the lipophilic, positively charged fluorescent dye 3,3′-dihexyloxacarbocyanine iodide (DiOC6). This dye binds to membranes (such as mitochondria and endoplasmic reticulum) and other hydrophobic, negatively charged cellular structures [26]. A decrease in the inner membrane potential, causing cells to become more electronegative compared to the control, results in increased dye absorption, indicating hyperpolarization. Conversely, an increase in membrane potential, making cells less electronegative, leads to dye expulsion, indicating depolarization [27]. In these assays, the mean fluorescence intensity (MFI) of control group cells measured at the 525 nm filter was set as 100% and used as the positive control. All experimental results were normalized relative to the control group data.

### 2.5. Statistical Analysis

The toxic effect was determined based on a statistically significant reduction in the number of microalgae cells in exposed samples compared to the control group. Statistical analyses were conducted using GraphPad Prism 8.0.2 (GraphPad Software, San Diego, CA, USA). The significance of the results was assessed using one-way ANOVA followed by Dunnett’s multiple comparisons test. The normality of residuals was verified using the Anderson–Darling test. A *p*-value of ≤ 0.05 was considered statistically significant.

## 3. Results

The mechanism of toxicity of different metal compounds is not always clear [28], as it may depend not only on the type of pollutant and its concentration [29] but also on its aggregate state and, in the case of nano- and microparticles, on their morphotype and size.

The use of microalgae as pollutant biosensors [30] is due to the fact that the susceptibility of microalgae to pollutants depends on several factors, including cell size, cell wall type and thickness, and taxonomic group characteristics [16].

In this work, the growth of the microalgae population under the influence of suspensions from underwater welding and cutting processes was considered as an evaluation parameter.

**Figure 1:**
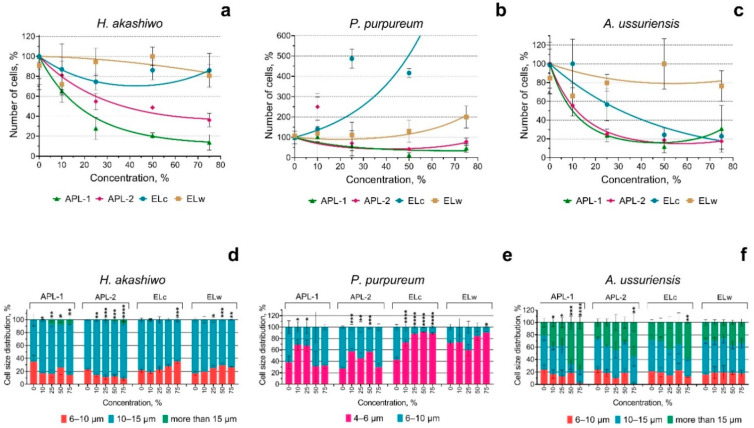
The growth rate inhibition and cell size distribution of three microalgae species after 72 h of the exposure to welding suspensions: (**a**–**c**) growth rate inhibition of *H. akashiwo*, *P. purpureum*, and *A. ussuriensis*, respectively; (**d**–**f**) cell size distribution of *H. akashiwo*, *P. purpureum*, and *A. ussuriensis*, respectively. *, *p* < 0.05; **, *p* < 0.005; ***, *p* < 0.0005; ****, *p* < 0.0001. 95% confidence limits presented by the whiskers.

Figure 1 shows the results of the evaluation of the effect of welding suspensions on the growth rate and change in cell size of three microalgae species after 72 h of exposure. The statistical significance of the effect of welding suspensions on microalgal growth rate (Figure 1a–c) was evaluated using ANOVA with Dunnett’s multiple comparisons test and is summarized in Appendix A.

The growth rate of the microalgae *H. akashiwo* and *P. purpureum* was most affected by APL-1 and APL-2 samples. Figure 1a,b shows the concentration-dependent inhibition of the growth rate of these two microalgae species. In microalga *A. ussuriensis*, in addition to samples APL-1 and APL-2, sample ELc also caused a concentration-dependent inhibition of growth rate. Sample ELw had no negative effect on the growth rate of any of the algal species. In red alga *P. purpureum*, samples ELc and ELw caused a concentration-dependent increase in growth rate.

The change in microalgae cell size was also most affected by samples APL-1 and APL-2 (Figure 1d–f).

The data correlate with studies by other researchers in the field of nanotoxicology; for example, exposure to zinc oxide nanoparticles (nZnO) inhibited the growth of *H. akashiwo*, and the toxic effect increased with increasing particle concentration and incubation time [31]. At high concentrations, the combined toxicity showed a synergistic effect, which may be related to the ‘Trojan horse effect’. This study is useful for understanding the effect of nanoparticles with different surface properties on marine algae growth.

**Figure 2:**
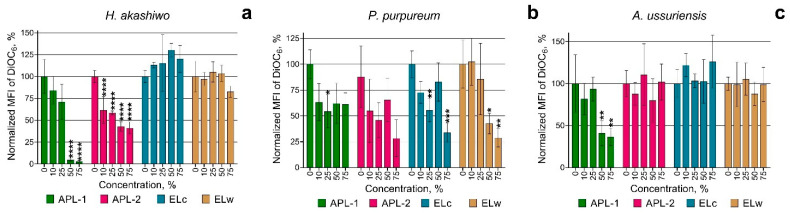
Membrane potential changes of three microalgae species after 72 h of the exposure to welding suspensions: (**a**) *H. akashiwo*; (**b**) *P. purpureum*; (**c**) *A. ussuriensis*. *, *p* < 0.05; **, *p* < 0.005; ***, *p* < 0.0005; ****, *p* < 0.0001. 95% confidence limits presented by the whiskers.

Figure 2 shows the change in membrane polarization of three microalgae species after 72 h exposure to welding suspensions.

When assessing changes in the membrane potential of microalgal cells, APL-1 and APL-2 samples caused significant concentration-dependent depolarization of *H. akashiwo* cells (Figure 2a). ELc and ELw samples did not induce any changes in the polarization of *H. akashiwo* cells. In diatom microalga *A. ussuriensis*, sample APL-1 also caused membrane depolarization at high concentrations (Figure 2c), whereas the other three samples of welding suspensions did not cause a statistically significant change in membrane polarization of *A. ussuriensis* cells. In contrast to *H. akashiwo* and *A. ussuriensis*, depolarization of the cell membranes of the red microalgae *P. purpureum* was only observed when exposed to ELc and ELw samples at the highest concentrations used (Figure 2b), which correlates with an increase in the growth rate of *P. purpureum* in these cases (Figure 1b).

## 4. Discussion

The results of this study highlight the importance of understanding the environmental risks associated with underwater welding and its impact on marine ecosystems [32]. The use of microalgae as biosensors to assess the toxicity of welding suspensions has revealed significant differences in the response of different microalgae species to micro- and nanoparticles generated during underwater welding. These differences may be related to the chemical composition of the particles, their size, and the peculiarities of the cell structure and physiology of the microalgae themselves.

### 4.1. Toxicity of Welding Suspensions

The most toxic to all three microalgae species were samples APL-1 and APL-2, which caused significant inhibition of growth rate and changes in cell size in all three microalgae species. This may be due to the high levels of heavy metals such as chromium (Cr), zinc (Zn), and copper (Cu) in these samples. These metals are known to be toxic to marine organisms, especially in the form of nanoparticles which have increased bioavailability and ability to penetrate cells. The results are consistent with other studies that also indicate the toxicity of metal oxide nanoparticles to marine organisms, especially at high concentrations [33].

Samples ELc and ELw showed less toxicity, which may be related to their chemical composition. However, it should be noted that these samples caused a concentration-dependent increase in the growth rate of the red microalga *P. purpureum*, which may indicate a stimulating effect at certain concentrations. This phenomenon requires further study as it may be related to adaptation mechanisms of the microalgae or to specific interactions of the components of the welding suspensions with the cell wall of this species.

### 4.2. Change in Membrane Potential

An important aspect of the study was the change in membrane potential of the microalgal cells. Samples APL-1 and APL-2 caused significant membrane depolarization in *H. akashiwo* and *A. ussuriensis*, indicating a disruption of cellular functions that may be related to the toxic effects of micro- and nanoparticles produced by underwater welding. In *P. purpureum*, depolarization was only observed when exposed to the ELc and ELw samples, correlating with an increase in the growth rate of this species. This may indicate a species-specific interaction between components of welding suspensions and cellular structures of microalgae of this species.

### 4.3. Environmental Impacts

These results highlight the need for further research to better understand the mechanisms of toxicity of micro- and nanoparticles produced by underwater welding. In particular, it is important to investigate the long-term effects of welding suspensions on marine ecosystems and to identify the most toxic metals and their combined effects. In addition, more research is needed on the ELc and ELw samples to find out how their components affect microalgae during long-term exposure.

It should also be noted that the manufacturers of the welding electrodes and wires used in the experiment claim the absence of chromium in their composition. However, our data show the presence of chromium in the welding suspensions, which may be due to its release from the St3sp steel plates during cutting and welding. This highlights the importance of controlling the composition of materials used in underwater operations to minimize environmental damage.

## 5. Conclusions

Overall, this study makes an important contribution to understanding the environmental risks associated with underwater welding and highlights the need to develop safer technologies and materials to minimize the impact on marine ecosystems. The results obtained can be used to develop recommendations for reducing environmental damage during underwater welding operations, as well as for further research in the field of nanotoxicology and ecotoxicology.

## Figures and Tables

**Table 1:** List of materials used for the study.

Sample Code	Welding Process	Mode Description	Dissolved Oxygen (O_2_), mg/L
APL-1	Flux-cored wire. Wet cutting	Automatic wet arc cutting with a 2 mm diameter PPR-APL1 flux-cored wire on DC polarity, at a current of 280–300 A, voltage of 37 V, with the torch position at an angle of 90 ± 15°, cutting speed of 180–200 mm/min. Two samples were taken 60 s after the start of cutting.	7.39 ± 0.11 *
APL-2	Flux-cored wire. Wet welding	Automatic wet arc welding with 1.6 mm diameter PPS-APL2 flux-cored wire (technical specifications 1274-001-83763787-2014) on reverse polarity current, at a current of 180 A, an arc voltage of 32 V, with the torch position at an angle of 90 ± 15° and a speed of 20 mm/min. A sample was taken after 60 s of welding.	8.59 ± 0.12 *
ELc	Coated electrode. Wet cutting	Manual wet arc cutting using Arcair size 5/16 × 14 (8.0 × 356 mm) electrodes, P/N: 42-059-007. A sample was taken 60 s after the start of cutting.	5.46 ± 0.11
ELw	Coated electrode. Wet welding	Manual wet arc welding using Arcair size 5/32 × 14 (3.97 × 356 mm) coated electrodes P/N: 42-984-004 on reverse current. A sample was taken after 60 s of welding.	6.15 ± 0.04 *

* Mean and standard deviation.

**Table 2:** Study of water by inductively coupled plasma mass spectrometry on an Agilent 7700× spectrometer (Agilent Techn., Palo Alto, CA, USA).

Element	ELw, ppb	ELc, ppb	APL-2, ppb	APL-1, ppb
Li	153	122	137	498
Mg	873,000	828,000	896,000	903,000
Al	309	280	8080	474
Ti	622	108	34.3	610
V	6.04	6.56	3.67	3.54
Cr	10.6	12.5	197	22.4
Mn	887	612	2480	1500
Fe	8440	20,700	43,100	12,300
Co	1.14	1.43	2.68	1.25
Ni	25.3	19.7	261	152
Cu	190	270	8710	2790
Zn	326	4100	1910	188
Ga	1.14	2.52	8.28	1.69
Ge	0.893	0.828	2.96	0.87
Se	2.02	1.21	1.38	2.16
Y	0.14	0.108	0.222	0.148
Zr	9.04	0.605	0.584	2.69
Nb	2.15	0.149	0.195	2.05
Ag	0.0779	0.0657	1.69	0.232
Cd	4.36	≤0.012	0.283	0.0872
Sn	11	50.8	41.4	4.97
Pb	7.41	4.95	160	10.9

**Table 3:** Bioassay endpoints and registration parameters.

Endpoint	Fluorescent Dye or Registered Parameter	Emission Channel/Band Width, nm	Dye Concentration for *H. akashiwo*	Dye Concentration for *P. purpureum*	Dye Concentration for *A. ussuriensis*
Growth rate inhibition	PI	610/20	15 µM	20 µM	15 µM
Cell size change	Forward scatter intensity (size calibration kit F13838 by Molecular Probes, USA)	FSC	–	–	–
Membrane potential change	DiOC_6_	525/40	2.5 µM	5 µM	1 µM

PI, Propidium iodide; DiOC_6_, 3,3′-dihexyloxacarbocyanine iodide.

## Data Availability

Data is contained within the article.

## Appendix A

**Table A1:** Significance of change in microalgae growth rate when exposed to seawater in which underwater welding was performed.

*H. akashiwo*								
Concentration	APL-1		APL-2		ELc		ELw	
	Summary	*p* Value	Summary	*p* Value	Summary	*p* Value	Summary	*p* Value
10	*	0.0147	ns	0.3119	ns	0.7163	ns	0.0550
25	****	<0.0001	**	0.0051	ns	0.2108	ns	0.9582
50	****	<0.0001	**	0.0019	ns	0.6740	ns	0.5088
75	****	<0.0001	***	0.0003	ns	0.6650	ns	0.4341
***P. purpureum***								
**Concentration**	**APL-1**		**APL-2**		**ELc**		**ELw**	
	**Summary**	***p* Value**	**Summary**	***p* Value**	**Summary**	***p* Value**	**Summary**	***p* Value**
10	ns	0.9979	**	0.0012	ns	0.9985	ns	0.9998
25	ns	0.1248	ns	0.7427	ns	0.1386	ns	>0.9999
50	***	0.0005	ns	0.2183	ns	0.3576	ns	0.9971
75	*	0.0258	ns	0.9029	****	<0.0001	ns	0.7750
***A. ussuriensis***								
**Concentration**	**APL-1**		**APL-2**		**ELc**		**ELw**	
	**Summary**	***p* Value**	**Summary**	***p* Value**	**Summary**	***p* Value**	**Summary**	***p* Value**
10	*	0.0225	**	0.0039	ns	0.9999	ns	0.3472
25	****	<0.0001	****	<0.0001	*	0.0302	ns	0.9781
50	****	<0.0001	****	<0.0001	***	0.0004	ns	0.5279
75	****	<0.0001	****	<0.0001	**	0.0021	ns	0.8829

*, *p* < 0.05; **, *p* < 0.005; ***, *p* < 0.0005; ****, *p* < 0.0001.
